# Sex differences in clinical characteristics, timeliness of care, and in-hospital outcomes of adult non-trauma patients in the emergency department

**DOI:** 10.1371/journal.pone.0332468

**Published:** 2025-09-17

**Authors:** Kun-Chuan Chen, Ji-Ze Hsu, Shu-Hui Wen

**Affiliations:** 1 Department of Emergency Medicine, Hualien Tzu Chi Hospital, Hualien City, Taiwan; 2 Institute of Medical Sciences, Tzu Chi University, Hualien City, Taiwan; 3 Department of Medical Research, Hualien Tzu Chi Hospital, Hualien City, Taiwan; 4 Department of Public Health, College of Medicine, Tzu Chi University, Hualien City, Taiwan; E-Da Cancer Hospital, TAIWAN

## Abstract

**Background:**

Data on sex differences in the clinical characteristics and outcomes of unselected emergency department (ED) patients are limited. We conducted a retrospective real-world cohort study to evaluate sex differences in clinical characteristics, ED timeliness of care, and in-hospital outcomes of adult non-trauma ED patients.

**Methods:**

Data from adult non-trauma patients who paid their first ED visit to a tertiary medical center from January 2018 to Jun 2020 were retrospectively analyzed. The patients were divided into male and female cohorts. The ED length of stay (LOS) was measured as the outcome of timeliness of care, whereas hospital admission, hospital LOS, and in-hospital mortality were measured as the in-hospital outcomes. Multivariate regression analyses were utilized to investigate the association between sex and outcomes.

**Results:**

Of the 43,661 patients included, 49.2% were males. The male cohort was older and had higher incidences of interhospital transfer and Taiwan Triage and Acuity Scale (TTAS) levels 1 and 2, higher mean Charlson comorbidity index, and more comorbidities than the female cohort. The male cohort had longer ED LOS and hospital LOS and higher incidences of hospital admission and in-hospital mortality. Multivariate regression analyses revealed that the male sex was an independent risk factor associated with adverse outcomes after adjustment for confounding factors. All these adverse outcomes were found in the male subgroup with TTAS levels 1–3.

**Conclusions:**

Our study identified sex differences in clinical characteristics, ED timeliness of care, and in-hospital outcomes of adult non-trauma ED patients. Male patients had various unfavorable conditions, including having older age, higher acuity levels, and more comorbidities, and were at higher unadjusted and adjusted risk for adverse outcomes on ED LOS, hospital admission, hospital LOS, and in-hospital mortality. The male subgroup with TTAS levels 1–3 was vulnerable to the negative impact of sex on these outcomes.

## Introduction

The emergency department (ED) is the frontline defense of any healthcare system and is a highly effective setting for patients’ urgent and lifesaving care [1]. ED clinicians perpetually endeavor to identify strategies for improving patient diagnosis and treatment. Sex has been recognized as an important factor that influences morbidity, mortality, pathogenesis, treatment response, and outcomes related to various diseases [2,3]. The sex differences can be caused by the effects of genetic predisposition on pathogenetic mechanisms, sex hormones, and environmental factors [2–6]. Thus, the incorporation of sex-based medicine in emergency medicine has important implications for the care and outcomes of ED patients [7,8]. A growing body of research on how sex influences clinical care and outcomes in ED patients has been conducted, but reporting of sex as an independent variable in emergency medicine research remains low [7,8].

Previous studies exploring sex differences in the clinical presentation, management, and prognosis of ED patients mainly focused on specialized conditions [9–23], such as acute coronary syndrome [10,11], stroke [12,13], and sepsis [21,22]. However, ED patients present with a vast array of disease conditions in real settings. Only limited data exist on sex differences in clinical characteristics and outcomes of unselected ED patients, and the findings remain inconclusive [24–27]. As such, more studies are required to address issues regarding sex differences in unselected ED patients.

We conducted a real-world retrospective cohort study to evaluate the sex differences in clinical characteristics, timeliness of care, and in-hospital outcomes of adult ED non-trauma patients in a tertiary academic medical center. Non-trauma patients were selected as the study cohort because they comprise the majority of ED visits and have various medical conditions [28,29]. Multivariate regression analyses were performed on data from ED patients who paid their first visit, and the association between sex and the outcomes was analyzed.

## Materials and methods

### Study design and setting

A retrospective cohort study of adult non-trauma ED patients admitted to Hualien Tzu Chi Hospital, Hualien, Taiwan, between January 1, 2018, and June 30, 2020, was conducted. The hospital is a tertiary academic medical center on the east coast of Taiwan and has an average number of ED visits of approximately 4000 per month. The ED has 22 universal patient care rooms and a 36-room observation unit. This study was approved by the Institutional Review Board of the hospital (approval number: IRB 109-214B). The informed consent was waived by the Institutional Review Board because of the retrospective study of medical records and the use of anonymized data. The date of access to the data for the purpose of this study is from 2/1/2021–3/1/2021. This study complied with the guidelines of the Strengthening the Reporting of Observational Studies in Epidemiology [30] and the Sex and Gender Equity in Research [31].

### Study participants

**Fig 1:**
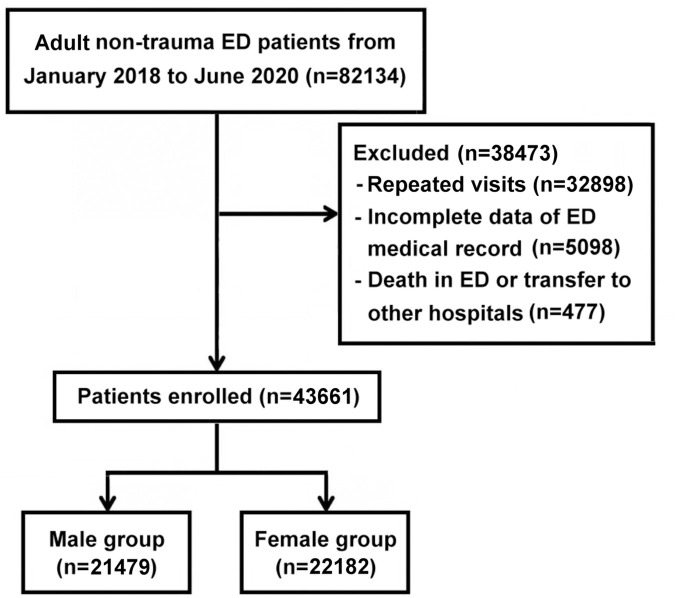
Flowchart of patient disposition. ED, emergency department.

We retrieved the data of the adult (age > 18 years) ED patients from the hospital’s electronic record system between January 1, 2018, and June 30, 2020. Data were reviewed independently by two abstractors who were blinded to the goal of this investigation. We identified a total of 82,134 ED visits of adult non-trauma patients after extracting de-identified patient data from ED visit records and inpatient medical files. Based on the triage records, patients’ chief complaints were categorized by the involved body systems. The distribution was as follows: gastrointestinal system (21.6%), nervous system (15.3%), cardiovascular system (10.5%), fever and infectious diseases (8.5%), ear/nose/throat (8.3%), musculoskeletal system (7.0%), respiratory system (7.0%), skin conditions (5.7%), urinary system (5.0%), eye conditions (2.4%), mental health (1.2%), endocrine disorders (0.7%), inter-hospital transfer patients (2.0%), and miscellaneous (4.8%). The variety of the involved body systems reflected a diverse patient population. For patients who visited the ED more than once during the study period, only data from the first visit were included. We excluded patients who had incomplete data in the ED medical records, who died in the ED, or who were transferred to other hospitals. Finally, a total of 43,661 patients were enrolled in this study and subdivided into male and female cohorts (Fig 1).

### Patients’ data measure

We collected demographic variables (sex and age), coexisting diseases, Charlson comorbidity index (CCI) scores, triage and acuity scales, and ED-related variables, which included year of ED visit, hour of ED visit, mode of arrival, specific treatments for time-sensitive diseases, and certain details after hospital admission. Sex was categorized as female or male according to the biological differences. Coexisting diseases were based on the International Classification of Diseases, 10th edition (ICD-10) diagnoses registered in the electronic health record. We used the five-level Taiwan Triage and Acuity Scale (TTAS) computerized system that was implemented nationally in 2010 to classify illness severity and prioritize patient care [32]. The five levels of TTAS are as follows: level 1, resuscitation; level 2, emergent; level 3, urgent; level 4, less urgent; and level 5, non-urgent. Specific treatments for time-sensitive diseases included antibiotics, anticoagulants, and inotropic agents. Admitting services subsequent to ED departure were categorized into non-intensive care units or intensive care units in medical and surgery departments.

### Outcome measures

We collected data on ED length of stay (LOS) as the outcome of timeliness of care. The ED LOS is an effective indicator of ED care quality [33] and can be divided into three subintervals: arrival to ED physicians, ED physicians to decision, and decision to departure. The durations of the subintervals were calculated using electronic health record timestamps. We also recorded hospital admissions, hospital LOS, and in-hospital mortality as the in-hospital outcomes.

### Statistical analysis

Kolmogorov–Smirnov test was used to check the normality of the distribution of the continuous variables. Continuous variables were compared using the independent sample t-test in the cases of normal distribution of data or the Wilcoxon rank sum test in the cases of non-normal distribution of data and are presented as mean ± standard deviation (SD) or median with interquartile range (IQR), respectively. The Chi-square or Fisher’s exact test was used in comparing categorical variables, which are presented as frequencies and percentages.

The association between sex and ED LOS or hospital LOS was investigated through multiple linear regression after controlling for confounders, including age, hours of ED visit, mode of transportation, TAAS level, and CCI. The results were reported as adjusted regression coefficients (β) with 95% confidence interval (CI). Log transformation was used to address skewed data for the analysis of ED LOS. Multivariable logistic regression was employed to evaluate the association between sex and hospital admission or in-hospital mortality after controlling for the same confounders; the results are reported as adjusted odds ratios (OR) with 95% CI. Similar multiple regression methods were used for additional subgroup analyses with patients stratified by different ages and TTAS levels. Bonferroni correction was adopted for correction for multiple testing in subgroup analyses. *p* < 0.05 was considered statistically significant. All analyses were conducted using SAS statistical software program version 9.4.

## Results

### Sex differences in patients’ characteristics

A total of 43,661 patients were enrolled in this study and subdivided into male (n = 21,479; 49.2%) and female cohorts (n = 22,182; 50.8%) (Fig 1). Table 1 shows the sex differences in the demographic and baseline clinical characteristics of patients between the cohorts. The male cohort had higher mean age and CCI as well as higher percentages of arrival via inter-hospital transfer and TTAS levels 1 and 2 than the female cohort. Meanwhile, the female cohort had higher percentages of the age group 18–44 years, off-hour visits, and arrivals through emergency medical services. The analysis of coexisting diseases revealed that the male cohort had higher percentages of several comorbid conditions, including myocardial infarction, peripheral vascular disease, cerebrovascular disease, diabetes with or without chronic complications, malignancies, leukemia, chronic kidney disease, chronic pulmonary disease, peptic ulcer, mild liver disease, and acquired immune deficiency syndrome or human immunodeficiency virus. Meanwhile, the female cohort had a higher percentage of rheumatic disease. Other between-group comparisons did not show statistical differences.

**Table 1:** Demographic and clinical characteristics of the study patients categorized by sex.

Characteristics	Male (n = 21479)	Female (n = 22182)	*p*-value
**Age, mean (SD)**	53.4 (19.5)	52.4 (20.7)	<0.001
**Age group, n (%)**			<0.001
18-44 years	7261 (33.8)	8323 (37.5)	
45-64 years	7536 (35.1)	6793 (30.6)	
≧65 years	6682 (31.1)	7066 (31.9)	
**Off-hour visit (6 pm-8 am), n (%)**	10591 (49.3)	11341 (51.1)	<0.001
**Mode of arrival, n (%)**			<0.001
Self-referred	17626 (82.1)	18355 (82.7)	
EMS	2490 (11.6)	2870 (12.9)	
Transfer	1363 (6.3)	957 (4.3)	
**Year of ED visit, n (%)**			0.246
2018	8531 (39.7)	8723 (39.3)	
2019	8929 (41.6)	9392 (42.3)	
2020	4019 (18.7)	4067 (18.3)	
**Level of TTAS, n (%)**			<0.001
1	967 (4.5)	599 (2.7)	
2	4900 (22.8)	4350 (19.6)	
3	14978 (69.7)	16554 (74.6)	
4	516 (2.4)	556 (2.5)	
5	118 (0.5)	123 (0.6)	
**Comorbidity**			
Charlson comorbidity index, mean (SD)	1.78 (1.8)	1.65 (1.8)	<0.001
**Cardiovascular disorders, n (%)**			
Congestive heart failure	590 (2.7)	654 (2.9)	0.216
Myocardial infarction	413 (1.9)	239 (1.1)	<0.001
Peripheral vascular disease	100 (0.5)	60 (0.3)	0.001
**Neurological disorders, n (%)**			
Cerebrovascular disease	1231 (5.7)	727 (3.3)	<0.001
Dementia	92 (0.4)	119 (0.5)	0.119
Hemiplegia or paraplegia	12 (0.1)	11 (0.0)	0.938
**Endocrine disorders, n (%)**			
Diabetes without chronic complication	1456 (6.8)	1335 (6.0)	0.001
Diabetes with chronic complication	394 (1.8)	343 (1.5)	0.022
**Hematologic and oncologic disorders, n (%)**			
Any malignancy	973 (4.5)	633 (2.9)	<0.001
Leukemia	70 (0.3)	45 (0.2)	0.016
Metastatic solid tumor	26 (0.1)	23 (0.1)	0.690
Lymphoma	23 (0.1)	19 (0.1)	0.570
**Renal and genitourinary disorders, n (%)**			
Chronic kidney disease	655 (3.0)	596 (2.7)	0.025
**Pulmonary disorders, n (%)**			
Chronic pulmonary disease	614 (2.9)	445 (2.0)	<0.001
**Gastrointestinal disorders, n (%)**			
Peptic ulcer disease	469 (2.2)	210 (0.9)	<0.001
Mild liver disease	416 (1.9)	319 (1.4)	<0.001
Moderate or severe liver disease	6 (0.0)	3 (0.0)	0.475
**Systemic rheumatic disorders, n (%)**			
Rheumatic disease	29 (0.1)	124 (0.6)	<0.001
**Infectious disorders, n (%)**			
AIDS/HIV	35 (0.2)	3 (0.0)	<0.001

EMS, emergency medical service; TTAS, Taiwan triage and acuity scale; AIDS/HIV, acquired immune deficiency syndrome/human immunodeficiency virus. Continuous and categorical variables are presented as mean with standard deviation (SD) and frequencies with percentage, respectively.

### Sex differences in ED timeliness of care and in-hospital outcomes

Table 2 shows sex differences in the ED timeliness of care and in-hospital outcomes of the patients. Analysis of ED timeliness of care revealed that the male cohort experienced longer ED LOS mainly because of the prolonged periods of physicians to decision and decision to departure, compared to the female cohort. In addition, the male cohort had higher percentages with regard to the use of antibiotics, anticoagulants, and inotropic agents and being admitted to the hospital. Analysis of admitting services subsequent to ED departure showed that the male cohort had higher incidences of admission to critical care in medical and surgical departments. Analysis of in-hospital outcomes revealed that the male cohort had a longer hospital LOS and a higher rate of in-hospital mortality.

**Table 2:** Emergency department care, disposition, and in-hospital outcomes of the study patients categorized by sex.

Characteristics	Male (n = 21479)	Female (n = 22182)	*p*-value
**ED timeliness of care (min),** **median [IQR]**			
Arrival to physician	7.0 [4.0, 12.0]	7.0 [4.0, 12.0]	0.974
Physician to decision	115.0 [57.0, 299.0]	109.0 [57.0, 243.0]	<0.001
Decision to departure	20.0 [8.0, 160.0]	15.0 [8.0, 88.0]	<0.001
ED LOS (min), median [IQR]	185.0 [87.0, 638.50]	155.0 [83.0, 441.0]	<0.001
**ED specific treatments to** **time-sensitive diseases, n (%)**			
Antibiotics usage	4242 (19.7)	3398 (15.3)	<0.001
Anticoagulant usage	1183 (5.5)	706 (3.2)	<0.001
Inotropic agent usage	611 (2.8)	401 (1.8)	<0.001
**Disposition, n (%)**			<0.001
Discharge	13547 (63.1)	16207 (73.1)	
Hospital admission	7932 (36.9)	5975 (26.9)	
**Admitting services, n (%)**			<0.001
Medical, non-critical care	4657 (58.7)	3965 (66.4)	
Medical, critical care	1236 (15.6)	625 (10.5)	
Surgery, non-critical care	1619 (20.4)	1165 (19.5)	
Surgery, critical care	420 (5.3)	220 (3.7)	
**In-hospital outcomes**			
Hospital LOS (day) (median [IQR])	6.0 [4.0, 12.0]	6.0 [4.0, 11.0]	<0.001
In-hospital mortality, n (%)	641 (8.1)	389 (6.5)	<0.001

ED, emergency department; LOS, length of stay. Continuous and categorical variables are presented as median with interquartile range (IQR) and frequencies with percentage, respectively. Data on in-hospital outcomes were analyzed using samples with hospital admission, while other data were analyzed using all samples.

### Association between sex and ED LOS or in-hospital outcomes

Table 3 shows the association between sex and ED LOS or in-hospital outcomes. Multivariate regression analyses revealed that the male sex was an independent factor associated with longer ED LOS and hospital LOS, as well as higher odds of hospital admission and in-hospital mortality compared to the female cohort after adjustment for confounding factors. Additional analyses with patients stratified by TTAS level revealed that all male sex–related adverse outcomes were found in the subgroups with TTAS levels 1–3, except that no sex difference in in-hospital mortality was observed in the TTAS level 3 group (Table 4). Additionally, the male subgroup with an age of <65 years was at higher adjusted risk for adverse outcomes, including longer ED LOS, longer hospital LOS, and higher odds of hospital admission. In addition, the male subgroup with an age of ≧65 years was at higher adjusted risk for a longer hospital LOS and higher odds of hospital admission and in-hospital mortality.

**Table 3:** Multivariate regression analyses of associations between sex and emergency department length of stay or various in-hospital outcomes.

Type of regression	Unadjusted	Adjusted^a^
Linear regression	*β* (95% CI)	*β* (95% CI)
ED LOS	0.16 (0.13, 0.18)*	0.10 (0.08, 0.12)*
Hospital LOS	0.06 (0.04, 0.09)*	0.07 (0.05, 0.10)*
Logistic regression	OR (95% CI)	OR (95% CI)
Hospital admission	1.59 (1.53, 1.65)*	1.61 (1.53, 1.68)*
In-hospital mortality	1.26 (1.11, 1.44)*	1.36 (1.19, 1.56)*

The female cohort was the reference group for analyses. ED, emergency department; LOS, length of stay; CI, confidence interval; OR, odds ratio; *, *p* < 0.05; ^a^, adjusted for age, off-hour visit, mode of arrival, level of Taiwan triage and acuity scale, and Charlson comorbidity index. Data on ED LOS and hospital admission were analyzed using all samples, while other data were analyzed using samples with hospital admission. Log transformation was used to address skewed data for the analysis of ED LOS and hospital LOS.

**Table 4:** Subgroup analyses of associations between sex and emergency department length of stay or various in-hospital outcomes stratified by different ages and triage levels.

	ED LOS		Admission		Hospital LOS		In-hospital mortality	
	Adjusted *β* (95% CI)	*p*-value	Adjusted OR (95% CI)	*p*-value	Adjusted *β* (95% CI)	*p*-value	Adjusted OR (95% CI)	*p*-value
**Age**								
<65 years	0.11	<0.001*	1.75	<0.001*	0.06	0.001*	1.25	0.069
	(0.08, 0.14)		(1.65, 1.86)		(0.02, 0.10)		(0.99, 1.58)	
≧65 years	0.03	0.221	1.33	<0.001*	0.07	<0.001*	1.34	<0.001*
	(−0.01, 0.08)		(1.23, 1.44)		(0.03, 0.10)		(1.14, 1.58)	
**Level of TTAS**								
4&5	0.01	0.856	1.26	0.293	−0.04	0.831	0.29	0.409
	(−0.12, 0.13)		(0.82, 1.94)		(−0.39, 0.31)		(0.01, 3.07)	
3	0.09	<0.001*	1.53	<0.001*	0.07	<0.001*	1.29	0.027
	(0.06, 0.11)		(1.45, 1.62)		(0.04, 0.10)		(1.03, 1.64)	
1&2	0.13	<0.001*	1.79	<0.001*	0.07	0.003*	1.38	<0.001*
	(0.08, 0.17)		(1.64, 1.96)		(0.02, 0.11)		(1.17, 1.63)	

The female cohort was the reference group for analyses. TTAS, Taiwan triage and acuity scale; ED, emergency department; LOS, length of stay; CI, confidence interval; OR, odds ratio; *, reached the significance level adjusted using the Bonferroni correction; ^a^, adjusted for age, off-hour visit, mode of arrival, TTAS level, and Charlson comorbidity index. Data on ED LOS and hospital admission were analyzed using all samples, while other data were analyzed using samples with hospital admission. Log transformation was used to address skewed data for the analysis of ED LOS and hospital LOS.

## Discussion

This real-world retrospective cohort study is one of the first studies that reported sex differences in clinical characteristics, timeliness of care, and in-hospital outcomes of adult non-trauma ED patients. We found several differences in clinical characteristics and outcomes between the sexes. The male cohort had worse clinical conditions in terms of age, incidence of interhospital transfer, TTAS level, CCI, and number of comorbidities. Moreover, the male cohort had poorer outcomes regarding ED LOS, hospital LOS, hospital admission, and in-hospital mortality. After adjusting for several confounders (age, hour of ED visit, mode of transportation, TAAS level, and CCI), we found that the male sex was an independent factor associated with these adverse outcomes. Further analysis revealed that all these adverse outcomes were found in the male subgroup with TTAS levels 1–3, suggesting that this subgroup was vulnerable to the negative impacts of the male sex on outcomes.

The findings of this study are clinically important because knowing sex differences in the clinical characteristics and outcomes of ED patients may help to establish sex-adjusted risk stratification tools, triage systems, and management strategies for ED patients [7,8,24]. It remains unclear how sex may influence the outcomes of our ED patients. One possible explanation is that the poorer outcomes observed in the male cohort are due to their unfavorable clinical conditions, particularly older age, high incidences of interhospital transfer, high TTAS levels, and high mean CCI. However, sex differences in outcomes in our patients cannot be explained by differences in these characteristics because the male sex remains an independent factor associated with poor outcomes after adjustment for confounders. The other explanation is that differences between males and females exist in the sex chromosomes, sex hormones, and environmental factors that are relevant to health and diseases [2–6]. For example, sex differences in disease pathogenesis, prevalence, manifestation, response to treatment, and outcomes are rooted in genetic differences between men and women [2,5]. Estrogens and androgens are known to differentially modulate the development and progression of diseases in males and females [4,5,34]. Also, there are a variety of environmental factors for which women and men differ that are directly linked to sex differences in disease predisposition and mortality [6,35,36]. All these sex-based disparities may have contributed to differences in the clinical characteristics and outcomes observed in this study.

The incorporation of sex-based medicine into emergency medicine has been recommended [7,8]. There is a growing interest in exploring the sex differences in the clinical characteristics, management, and prognosis of ED patients. However, this topic has mostly been investigated in small, diagnosis-based subpopulations with a focus on specialized conditions [9–23]. Most of these studies found sex differences in the clinical characteristics and in various outcomes of interest [9–11,13–15,17–20] that favored male [9,10,13,14,17,19,20] or female patients [9,11,15,19,20]. However, data from these studies are difficult to generalize to real-world ED settings because ED patients present with a vast array of diseases. However, there is scant research investigating this topic in unselected ED patients. Candel et al. [24] enrolled 148825 adult ED patients (females, 48.8%) and reported that, although patient characteristics for both sexes were comparable, males were at higher adjusted risk for in-hospital mortality and intensive care unit/medium care unit admission. Thomas et al. [25] enrolled 34,333 adult internal ED patients (females, 51.2%) and showed that their male patients were older and had more risk factors (e.g., smoking, diabetes, and hyperlipoproteinemia) than female patients. Their univariate analyses [25] revealed that no significant sex differences in hospital LOS and in-hospital mortality were found, but men were at higher unadjusted risk for admission for inpatient and intensive care treatments. Cournane et al. [26] enrolled 58126 ED patients (females, 51.2%), did not compare clinical characteristics between sexes, and reported that 30-day in-hospital mortality adjusted for outcome predictors was similar for males and females. Onal et al. [27] enrolled 64,117 ED patients (females, 51.7%) and showed that patient characteristics for both sexes were comparable but the males had a higher percentage of emergency severity index level 2. Particularly, their multivariate analyses [27] revealed that female patients had slightly longer ED LOS, door-to-room, and door-to-healthcare practitioner than males, but these differences did not meet their threshold for clinical significance. Collectively, these studies suggested that clinical characteristics and outcomes may or may not be different between sexes. Whether sex is a risk factor for the outcomes of unselected ED patients remains inconclusive. Similar to these studies, this study also enrolled unselected non-trauma patients. However, we extensively analyzed several metrics of ED timeliness of care and in-hospital outcomes in the same real-world cohort. As such, our findings may provide valuable information that can advance our knowledge regarding this topic.

In this study, we found that male non-trauma ED patients were older, had more severe illness, and more comorbidities than females. After adjustment, male sex remained an independent risk factor for worse outcomes, especially in higher severity cases (TTAS 1–3). These findings suggest that even after adjusting for age, severity, and comorbidities, male ED patients still had unfavorable outcomes. This may be related to potential biological differences (e.g., immunity, hormones, inflammatory responses), differences in health behaviors, or unmeasured confounders (e.g., social support and lifestyle). From the clinical perspective, our findings emphasize the importance of thorough evaluation, comprehensive comorbidity management, patient education, close follow-up, and psychosocial support to improve outcomes in this vulnerable subgroup. Also, from a policy perspective, male sex could be considered a high-risk alert to prompt more rigorous clinical assessments (e.g., more intensive vital sign monitoring, early treatment evaluation). Future research is warranted to investigate the mechanisms underlying the sex differences in ED outcomes.

Our hospital is the only tertiary medical center on the east coast of Taiwan, providing medical care covering a longitudinal valley area with a scattered population. The study period (January 2018 to June 2020) was determined based on the timeline of our research project, and the data cutoff was related to the schedule for project writing and review processes. Although the COVID-19 pandemic began in 2020 in Taiwan, the outbreak was well-controlled through strict and prompt public health measures. By the end of June 2020, there were only around 500 cumulative confirmed cases in Taiwan [37], and no significant impact on emergency department visit volumes or disease patterns was observed. Therefore, we believe that the influence of COVID-19 on our study results during this period was minimal, and this timeframe can be considered to represent the pre-pandemic emergency department status. A key strength of our study is its large‐scale analysis using real‐world data from routine clinical practice. However, several limitations need to be considered. First, our study was designed to analyze preexisting data and is subject to several biases, including data collection from the medical records of patients and the inherent differences between the male and female cohorts. Some differences between them may have not been measured and may have accounted for our observed results. Second, our participants were non-trauma patients who visited the ED of a single medical center. Thus, our results cannot be generalized to all ED patients or all hospitals. Third, findings from our subgroup analyses should be interpreted with caution because stratification results in limited statistical power. Fourth, our data were retrieved from the hospital’s electronic record system and may have been subjected to errors in documentation and data entry. Fifth, we identified males and females between 2018 and 2020 and excluded repeated visits. These data evidently did not represent the first ED visit in their lifetime. However, the study cohort may provide valuable information in a real-world setting.

## Conclusion

Our study identified sex differences in clinical characteristics, timeliness of care, and in-hospital outcomes of adult non-trauma ED patients. Male patients had several unfavorable conditions, including having older age, higher acuity levels, and more comorbidities, and were at higher unadjusted and adjusted risk for adverse outcomes on ED LOS, hospital admission, hospital LOS, and in-hospital mortality. Further investigations regarding the potential application of the detected sex differences in the risk stratification and management of ED patients are warranted.
